# Scale and structure dependent solute diffusivity within microporous tissue engineering scaffolds

**DOI:** 10.1007/s10856-020-06381-x

**Published:** 2020-05-04

**Authors:** Giovanni S. Offeddu, Lakshana Mohee, Ruth E. Cameron

**Affiliations:** Cambridge Centre for Medical Materials, Department of Materials Science and Metallurgy, University of Cambridge, 27 Charles Babbage Rd, Cambridge, CB3 0FS UK

**Keywords:** Porous media microstructure, Cell microenvironment, Collagen, FRAP, Molecular transport

## Abstract

Diffusion of nutrients to cells cultured within three-dimensional scaffolds is fundamental for cell survival during development of the tissue construct, when no vasculature is present to aid transport. Significant efforts have been made to characterize the effect of structure on solute diffusivity in nanoporous hydrogels, yet a similar thorough characterization has not been attempted for microporous scaffolds. Here, we make use of freeze-dried collagen scaffolds, possessing pore sizes in the range 150–250 μm and isotropic or aligned morphology, to study the diffusivity of fluorescent dextran molecules. Fluorescence recovery after photobleaching is used to measure the self diffusivity of the solutes within single pores, while Fickian diffusion over scales larger than the pore size is studied by assessing the solute concentration profile within the materials over time. We show that, not only do the morphological parameters of the scaffolds significantly affect the diffusivity of the solutes, but also that the assessment of such diffusivity depends on the length scale of diffusion of the molecules under investigation, with the resulting diffusion coefficients being differently affected by the scaffold structure. The results provided can guide the design of scaffolds with tailored diffusivity and nutrient concentration profiles.

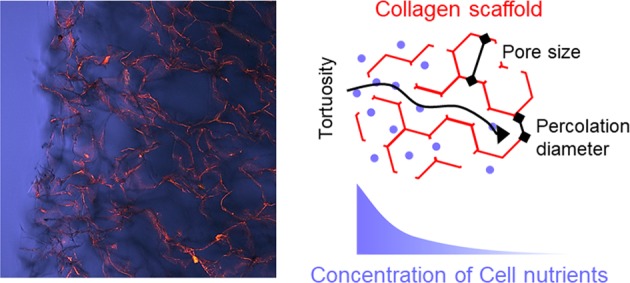

## Introduction

Tissue engineering scaffolds have evolved in the last two decades to recapitulate the cellular microenvironment found in a variety of tissues. The design variables available for scaffolds are numerous, even after satisfaction of fundamental requirements such as the use of non-cytotoxic materials and porous, hydrated morphologies [1]. Indeed, cell substrates can be made to be nanoporous to encapsulate cells in a rich biomimetic extra-cellular matrix (ECM) environment, as in the case of most hydrogels [2], or microporous, in order to facilitate three-dimensional (3D) cell seeding and migration [3] to then rely on the cells to produce the natural ECM. The ability of solutes, cell nutrients in particular, to move through these structures is of paramount importance to ensure homogeneous cell viability during development of the tissue construct, as the lack of vasculature results in cells being farther away from the source of nutrients compared with in vivo conditions [2]. Accurate assessment of scaffold transport properties is, therefore, necessary to ensure successful translation to clinical settings.

Solutes reach cells in interstitial spaces predominantly by diffusion, i.e. as the result of concentration gradients [4]. The capacity of a particular solute to move by diffusion, translating from large to small concentrations, is quantified by its translational, or Fickian, diffusion coefficient, *D*. The local random movement of the solute is characterized by the self diffusion coefficient, *D*_S_, a quantity understood to be numerically equal to *D* in the absence of concurring thermodynamic effects increasing solute translation [5]. Within nanoporous hydrogels, diffusivity has been investigated in order to understand how the mobility of solutes is affected by the polymer network, whose morphology can be described by one parameter only, the pore size, as this is always proportional to the solid volume fraction [6]. Fluorescence recovery after photobleaching (FRAP) was used in several studies to measure *D*_S_ for molecules of comparable size to the nanopores of the hydrogels, revealing a sieving effect by the polymer network [7–10]. Microporous scaffold materials, instead, are defined by structural features that are orders of magnitude larger than molecules (tens to hundreds of microns compared to nanometers), which determine bulk properties vastly different from those of hydrogels [11]. These structures may possess more complex geometries that cannot be described only by the pore size, as often the pores are partially occluded so that the effective size and tortuosity of the transport paths may be different for scaffolds of comparable pore size or solid fraction [11]. Therefore, it is hypothesized here that it is not only the pore size or solid fraction that influences the response [12, 13], and that further structural parameters will affect solute diffusion in microporous materials.

Freeze-dried collagen scaffolds represent an ideal platform to measure diffusional phenomena in controlled microscale morphologies. The materials are produced through ice-templating of a collagen slurry, where control over the freezing rate and resulting ice nucleation and growth results in isotropic structures with custom pore size and interconnectivity [3, 14–16]. Directional freezing of the slurry can also be used to fabricate aligned structures that allow for greater cell penetration and, as we have recently shown, larger permeabilities to interstitial fluid flow [17]. Well-established characterization techniques can be applied to measure and compare the structural parameters describing the morphologies created [18]. Importantly, these materials were used in the pioneering study on tissue engineering by Yannas and coworkers [19], and have since been employed in several clinical trials attesting to their clinical potential [20, 21].

In this study, we make use of microporous freeze-dried collagen scaffolds possessing isotropic and aligned morphologies, and characterized by varying structural parameters such as pore size, percolation diameter, tortuosity, and solid volume fraction. The slurry concentration used to form the ice templated scaffolds is varied, giving structures with different architectures and interconnectivities. The scale-dependent diffusivity of solutes is investigated using dextran molecules of varying molecular weight as models for physiologically-relevant proteins. Diffusion over scales larger than the characteristic pore size is assessed using a simple experimental set up to measure the translational diffusion coefficient into the materials; FRAP is used to measure the local self diffusion properties within the pores of the structures. The results reported confirm the importance of scaffold structure for solute diffusivity, and ultimately for the effective use of these materials as 3D cell culture environments.

## Materials and methods

### Materials

Collagen (insoluble fibrillar type I from bovine Achilles tendon), 1-ethyl-3-(3-dimethylaminopropyl) carbodiimide hydrochloride (EDC), n-hydroxysuccinimide (NHS), rhodamine B, and fluorescein isothiocyanate (FITC)-conjugated dextrans of molecular weight 4, 20, 250, and 2000 kDa, were all purchased from Sigma Aldrich UK and used without further purification. Phosphate buffered saline (PBS) was purchased from Thermo Fisher UK.

### Scaffold fabrication

Scaffolds were fabricated with type I bovine collagen using a well-established method detailed in [22]. Suspensions of collagen in 0.05 M acetic acid were made at a solid concentration of 0.5, 0.75, and 1% w/V. Blending and centrifugation (2500 rpm, 15 min) produced bubble-less homogeneous slurries, which were poured in polystyrene six-well plates to form isotropic structures, and in custom-built molds made with polycarbonate and a stainless steel base to form aligned structures through the application of a directional thermal gradient. Freeze-drying was carried out in a VirTis adVantage bench-top freeze-drier (Biopharma Process Systems, UK), using a cooling rate of 0.5 °C min^−1^ from room temperature down to a temperature of −20 °C, which was maintained for 2 h. In the case of the aligned structures, the freeze-drier shelf and molds were pre-cooled to the freezing temperature (−20 °C) before the start of the process. A vacuum of 80 mTorr was then applied at 0 °C to dry all samples over 20 h. Crosslinking of the scaffolds was performed using EDC and NHS, both in 95% V/V ethanol in water at a molar ratio of 5:2:1 to the collagen carboxyl groups (EDC: NHS: Carboxyl). The samples were finally washed in water three times for 5 min and freeze-dried again as described above.

### Measurement of translational diffusion coefficients

The sides of the collagen scaffolds were cut off using a scalpel to eliminate any external skin where the structure may be denser than the bulk of the materials [22]. The sections obtained, approximately 1 cm × 5 mm × 3 mm, were placed in a solution of 0.1 mg mL^−1^ rhodamine B in distilled water and de-gassed for 20 min at 1200 mTorr in the freeze-drier, and thus stained overnight. The same samples were then washed in PBS three times to ensure removal of any non-bound rhodamine B.

**Fig. 1:**
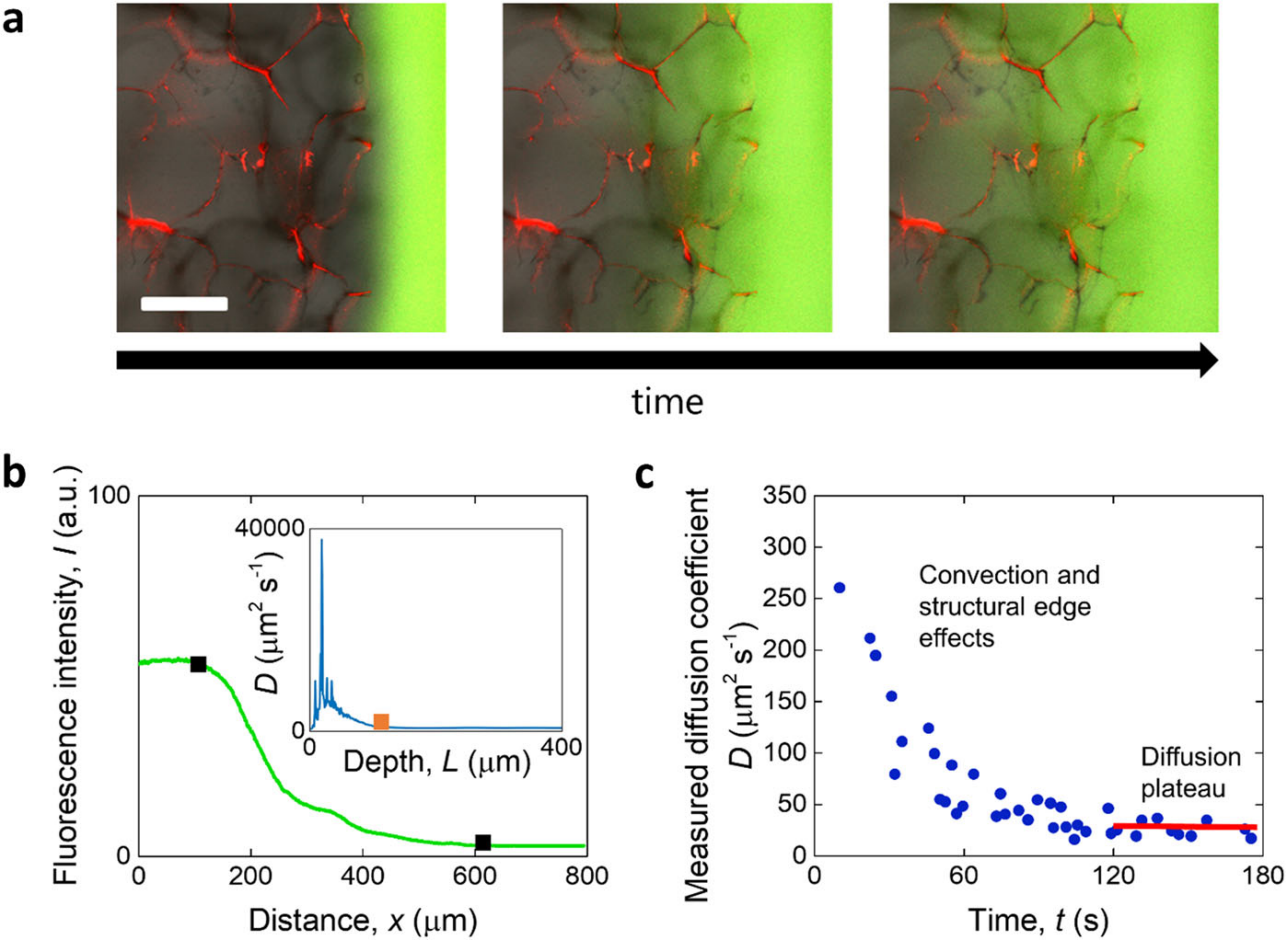
Measurement of translational diffusion coefficient. **a** Translation of dextran into microporous collagen scaffold with time, observed by confocal microscopy. The scale bar is 200 μm. Green: FITC-dextran; red: rhodamine-stained scaffold. **b** Example fluorescence intensity profile with distance. The black squares represent the surface of the scaffold (left) and the end of the translating dextran front (right), respectively. In the inset, a typical profile of the diffusion coefficient with depth into the sample is shown, where the orange square represents the point after which the coefficient is averaged. **c** Time-dependence of the measured translational diffusion coefficient with time where two regions are observed whereby large values determined by convection and edge effects reduce to a stable plateau determined by diffusion only

Solutions of FITC-dextran were prepared fresh in PBS at a concentration of 0.1 mg mL^−1^. Imaging was performed on an Olympus FV1200 confocal microscope (Olympus, JP) at room temperature using two lasers, 488 nm and 543 nm, with a 10X objective. Hydrated scaffold samples were placed on a glass slide and provided with additional PBS (approximately 100 μL) to ensure swelling saturation and to form a fluid environment surrounding the solid scaffolds. Using a pipette, a 50 μL droplet of FITC-dextran solution was gently put in touch with the fluid around the sample, and a recording was started in which the confocal microscope took images of the side of the sample closer to the droplet every ≈2 s. The FITC-dextran front appeared in the field of view within several frames and was recorded to reach the sample surface (imaged through the rhodamine B signal) to then slowly move into it over time, as shown in Fig. 1a.

Analysis of the translational diffusion coefficient *D* was performed in MatLab (MathWorks, US) for those time points where the fluorescent solution was in contact with the samples: At each time-point, the fluorescence intensity profile was plotted for the entire field of view. Sign-change analysis was used to automatically identify the surface of the sample, i.e. the point at which the intensity began to decrease compared with the outside fluid, and the background depth, i.e. the point at which the intensity stopped decreasing and stabilized to a non-zero value (Fig. 1b). The segment of the intensity profile contained within the sample surface and background was compared to that for the previous time point (i.e. the previous intensity at each depth was subtracted from the later one), and the instantaneous diffusion coefficient was calculated using a common derivation of Fick’s second law for a constant source [23], as previously reported for diffusion within the brain interstitium [24], using:

1 $$\frac{{I_L}}{{I_0}} = erfc\left( {\frac{L}{{2\sqrt {Dt} }}} \right)$$

where *I*_L_ is the fluorescence intensity at a depth *L*, *I*_0_ is the average intensity at the source, that is the fluorescence intensity outside the sample, and *t* the time between time-points. The assumption was made that fluorescence intensity scales linearly with solute concentration, as done previously [24–26].

A typical profile of *D* with depth is shown in the inset of Fig. 1b: Up to a depth of approximately 100 μm, the translational diffusion coefficient was non-constant and showed spikes to very large values, to then fall to a smaller near-constant plateau value for the remaining depth. The variability at small distances may result from surface effects arising from partial pores at the edges. Using sign-change analysis again, *D* was taken as the average of the plateau smaller values for each time point. As the last step, *D* was plotted as a function of time (Fig. 1.c) and always found to decrease within several frames at short times, to then stabilize to a plateau at longer times. This trend is likely to be due to transient convective transport resulting from the additional fluid on one side of the material, which increases the apparent value of *D* at short times [27]. In this work, the value of the translational diffusion coefficient *D* was approximated as that measured at *t* = 150 s, at which time the trend had stabilized to a plateau value in all cases. By calculating *D* in this way for all scaffolds and all probe molecules, it was possible to explore the effect of these parameters on transport.

### Measurement of self-diffusion coefficients

The self diffusion coefficients, *D*_S_, of FITC-dextrans within the various scaffold structures fabricated were assessed by FRAP. The samples were submerged in FITC-dextran PBS solutions (same concentration as above) and then imaged on the confocal microscope using the 488 nm laser. The same laser was used to bleach a circular spot within pores of the samples over ≈2 s, followed by a recording of the fluorescence recovery every 67 ms for up to ≈ 25 s. The spot size was varied between 5 and 180 μm to probe any change in diffusivity measured, and set to 30 μm for the rest of the experiments. Analysis of the recovery profile was performed on FRAPAnalyser (University of Luxembourg, ActinSim), which applies the formula [28]:

2 $$D_{\mathrm{S}} = w^2/t_{\mathrm{R}}$$

where *w* is the radius of the circular spot and *t*_R_ the recovery time calculated by the software.

### Structural analysis

A standard set of samples was used for the experiments reported here as in our study on the permeability to fluid flow of these materials [17]. The structural parameters describing the samples were obtained through analysis of tomography data. In brief, the collagen scaffolds were imaged using an X-ray micro-tomography scanner (Skyscan 1172, Bruker, UK). The optimized settings used a voltage of 25 kV and a current of 137 μA. The 3D reconstructed images, corresponding to each scaffold architecture, were analyzed using ImageJ, distribution FIJI, in terms of collagen volume fraction, structure average pore size, percolation diameter, and tortuosity. Each dataset was initially binarized using the Trainable Segmentation plugin, part of the ImageJ software. Noise removal was carried out using both the Despeckle function and the Median 3D filter. All outliers larger than 2 pixels were filtered out, and the volume fraction of collagen, *φ*_c_, was measured directly as the percentage of volume occupied by solid. The Watershed algorithm was used to fit each pore with an ellipse, and the pore size *d* was calculated from the average diameter of each ellipse. The average pore aspect ratio for the aligned scaffolds was calculated from the fraction of the two orthogonal ellipse diameters, denoted *a* (shorter diameter) and *b* (longer diameter) fit to the pore sections orthogonal to the direction of diffusion. The pore aspect ratio was assumed to be unity for the isotropic scaffolds.

Percolation theory was used to calculate the percolation diameter of the porous media. This mathematical analysis reveals the interconnectivity of pores within the scaffolds, by determining the accessible lengths of a range of particle sizes within the porous structure [18]. The percolation diameter was obtained by applying the “Shrink Wrap” function to the binarized datasets. Using the results, the percolation diameter, *d*_p_, was calculated as the y-intercept of the following relationship [29]:

3 $$L = L_0\left( {\delta - d_p} \right)^{ - \upsilon }$$

where *δ* is the particle diameter, *L* is the depth traveled by the particle within the structure, *ν* is equal to 0.88 in 3D, and *L*_0_ is a constant. The percolation diameter, in the case of the aligned scaffolds, was measured in the direction of alignment.

Tortuosity can be defined as the ratio of the effective path between two points to the Euclidean distance between start and end of the path [30]. It was mathematically calculated using the Skeletonize option in ImageJ. This algorithm connects all voxels that represent the pore structure to form branch lengths. The skeletons were then analyzed by the software and the tortuosity, *τ*, calculated.

### Statistical analysis and data representation

The translational diffusion coefficient was measured for five samples per condition (scaffold morphology and specific solute), while ten FRAP measurements were conducted on the same five samples per condition to measure the self diffusion coefficient. All data is reported as mean ± standard deviation. Model fitting and statistical analysis were performed using the software OriginPro 2016, where a probability *p* of 0.05 was taken as the threshold value to ascertain statistically significant differences in two-sample *t*-tests.

## Results

Structural studies were conducted on the different scaffolds fabricated (Table 1). As the concentration of collagen in the slurry was increased, the final scaffold structure varied significantly. In particular, the volume fraction of collagen increased with slurry concentration, and the pore size and percolation diameter decreased. At the same time, the tortuosity of the porous structure increased with collagen concentration in the slurry. Compared with the isotropic structure made from the same slurry concentration (0.75% w/V), the aligned structure presented a larger final solid fraction and smaller pore size, but larger percolation diameter and smaller tortuosity.

**Table 1:** Structural parameters of freeze-dried collagen scaffold as a function of collagen concentration in the slurry and the application of uniform or directional freezing profiles [16]. For the aligned scaffolds, *a* and *b*, representing the shorter and longer radii of the ellipses fit to the pores, were measured as 85 ± 24 μm and 485 ± 130 μm, respectively

Scaffold condition	*d* (μm)		*d*_p_ (μm)		*φ*_c_ (%)		*τ*	
	Average	Std. dev.	Average	Std. dev.	Average	Std. dev.	Average	Std. dev.
0.5%	221	22	187	10	0.58	0.13	1.34	0.11
0.75%	187	28	124	18	0.65	0.06	1.58	0.18
1%	165	15	89	19	1.25	0.13	1.74	0.25
Aligned	160	6	143	3	0.83	0.11	1.43	0.15

**Fig. 2:**
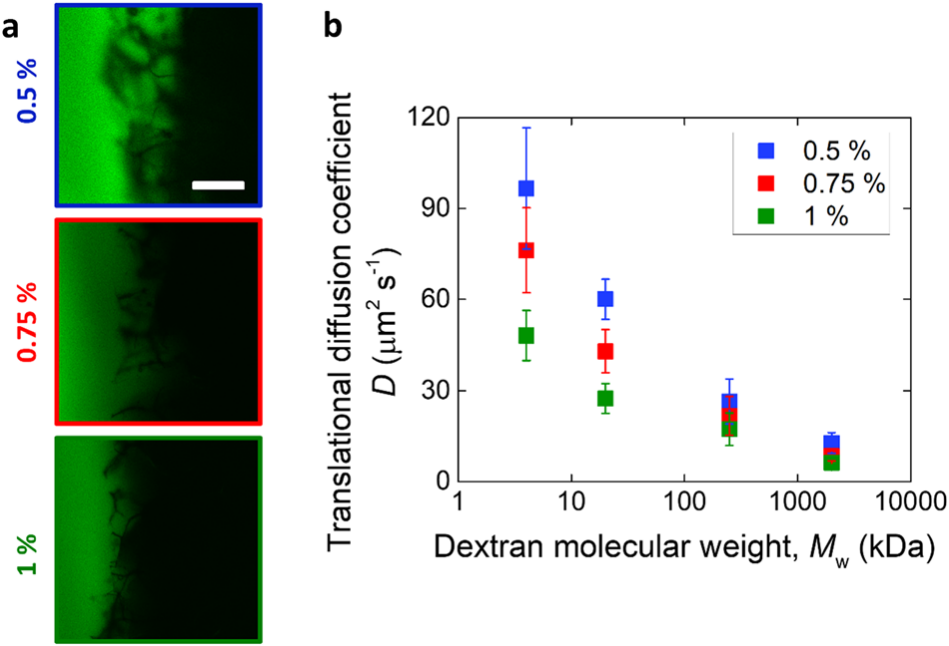
Translational diffusion in collagen scaffolds. **a** Representative confocal microscopy images of dextran solutions in contact with scaffolds of varying slurry concentrations for 60 s. The scale bar is 200 μm. **d** Translational diffusion coefficient for scaffolds made with varying slurry concentration, as a function of dextran molecular weight

The results of the analysis for the translational diffusion coefficient in the isotropic structures of dextrans with varying molecular weight are reported in Fig. 2. Qualitatively, the fluorescence front was observed to reach a farther depth with time within the scaffolds characterized by a smaller collagen concentration used in their slurries (Fig. 2a). Measurement of the diffusion coefficient, reported in Fig. 2b, indeed showed *D* decreasing with collagen slurry concentration (*p* < 0.05 between 0.5% w/V and 1% w/V), as well as dextran molecular weight (*p* < 0.05 in all cases).

**Fig. 3:**
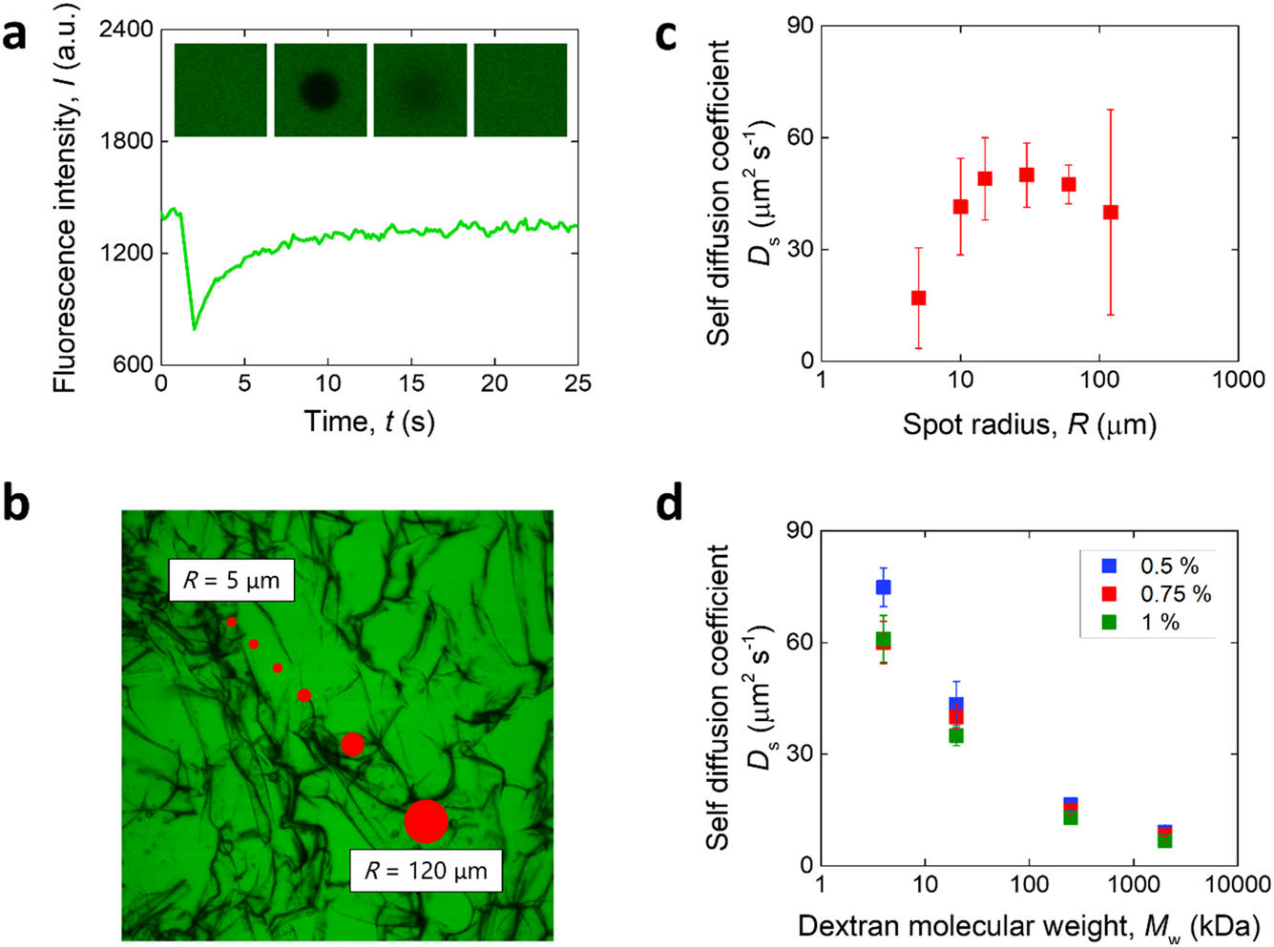
Measurement of self-diffusion coefficient. **a** Representative fluorescence intensity profile with time, recorded during a FRAP experiment. The insets show the area around the bleached spot varying with time. **b** Comparison between spot sizes and microstructure of a 0.75% collagen scaffold (dark) filled with dextran solution (green). **c** Self diffusion coefficient versus spot radius for a 0.75 % collagen scaffold. **d** Self diffusion coefficient of dextran in microporous collagen scaffolds as a function of dextran molecular weight

FRAP was used to assess the self diffusion coefficient of the same solutes within the scaffold structures. A spot was bleached within single pores of the scaffolds, and the shape of a typical fluorescence recovery profile is displayed in Fig. 3a. The spot size was varied between 5 and 120 μm, compared in Fig. 3b to a 0.75% w/V structure. The resulting *D*_S_ for the structures is shown in Fig. 3c as a result of such variation in spot size. It was found that the average self diffusion coefficient initially increased with spot size to reach a fixed value, yet the variability in the measurement increased as the spot size became larger than 100 μm. Recovery data for spots larger than 120 μm (not shown) was too noisy to be successfully analyzed by the software used. A spot size of 30 μm was chosen for further analysis. The resulting self diffusion coefficients for the dextrans in the isotropic structures are reported in Fig. 3d. As for the translational diffusion coefficient results, *D*_S_ decreased with dextran molecular weight and, on average, with increasing collagen slurry concentration (*p* < 0.05 between 0.5% w/V and 1% w/V), although the difference was much less marked.

**Fig. 4:**
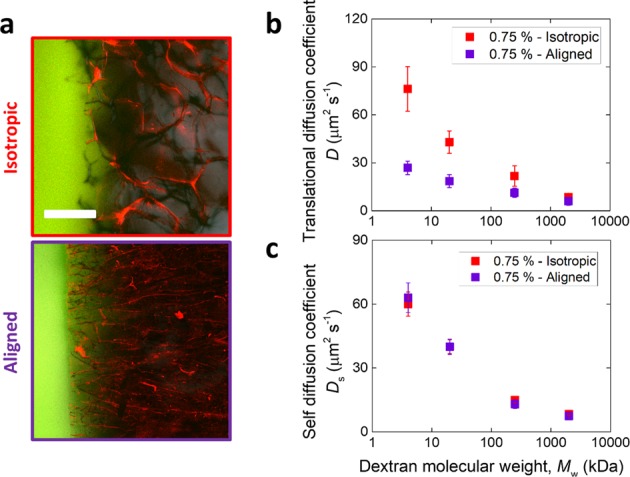
Comparison between isotropic and aligned scaffolds. **a** Confocal images of isotropic and aligned collagen scaffolds (red) in contact with dextran solutions (green). The scale bar is 200 μm. **b** Translational and **c** self -diffusion coefficient of dextran in both structures as a function of dextran molecular weight

Comparison between isotropic and aligned structures was made both in terms of translational and self diffusion. Figure 4a shows the qualitative difference between the two, whereby larger, non-aligned pores can be seen in the former, while aligned pores perpendicular to the outside solution are visible in the latter. Both translational (Fig. 4b) and self (Fig. 4c) diffusion coefficients decreased with dextran molecular weight, with the average always larger for the isotropic structures in the case of translational diffusion (*p* < 0.05), but comparable for the two structures for self diffusion (*p* > 0.05). All translational and self diffusion coefficients measured as part of this study for the various scaffold conditions are summarized in Supplementary Table 1 and Supplementary Table 2, respectively.

## Discussion

The clinical translation of tissue engineered scaffolds has suffered, in part, from the ineffective transport of cell nutrients and oxygen within large constructs, resulting in different tissue formation patterns in the center compared with the edges of the scaffolds [31, 32]. Understanding the factors that can affect diffusion in microporous scaffolds can help to address this issue, while also providing insight into the use of these materials in other biomedical applications, such as in drug delivery [33, 34] or as part of bioreactors [35, 36]. The study of dextran diffusivity in microporous collagen scaffolds conducted here, in particular, shines light on a number of phenomena that improve our understanding regarding the use of these and other microporous materials in such applications.

Diffusion is the predominant mechanism by which cells in vivo receive nutrients from the vasculature, playing a major role in ensuring cell viability with depth into the scaffold during tissue culture in vitro [37–39]. Our assessment of the translational diffusion coefficient within microporous materials showed that this parameter apparently varies with depth, decreasing to a plateau value at depths greater than a distance approximating the pore size (≈100 μm, Fig. 1a). The behavior could be explained by considering that the superficial pores, first to oppose the movement of the dextran, are sectioned and so partially open to the outside solution, therefore limiting the solute mean free path to a lesser extent than in the bulk of the scaffold. It is only when the solutes reach greater depths into the scaffold that the constraining effect of the structure begins to dominate their diffusivity. The large *D* values measured at shallow depths may also be due to convection near the surface, a phenomenon expected [40] and visualized for all time points.

Significant morphological differences in the isotropic scaffolds utilized for the studies were produced by varying the concentration of collagen in the slurry. In fact, as the slurry concentration was increased, so was the concentration of collagen present in the final scaffold (Table 1). A larger volume fraction of collagen in these materials results in more of the pore surface becoming covered by pore walls [11]. As a result of this effect and the decreasing pore size with concentration, the percolation diameter, i.e. the size of the transport paths, also decreased, while the tortuosity of such paths increased. Interestingly, these structural variations were found to affect the translational and self diffusivity of the dextrans differently, decreasing the mobility of the molecules more predominantly over scales larger than the pore size (i.e. those assessed by the translational diffusion coefficient, rather than the self diffusion coefficient, Fig. 2b compared to Fig. 3d).

Indeed, self diffusivity of dextrans within the microporous structures could only be studied at a scale smaller than the pore size. FRAP was used for this purpose, a technique that measures the self diffusion coefficient *D*_S_ by considering the fluorescence recovery within a bleached spot, as previously done in nanoporous materials such as hydrogels [6–8]. The size of the spot in those hydrogel studies was larger than the pore size of the materials, so that the effect of structure on the diffusivity could be ascertained. Here, the spot size was changed to observe any effect on the measured self diffusion coefficient (Fig. 3). It was found that a small spot size (5 μm) resulted in a smaller value of *D*_S_, yet for values up to 60 μm the diffusion coefficient measured was constant. A small spot size may result in non-uniform bleaching, which in turn compromises the analysis [41]. Large spot sizes could also not be used: when the size was set as 120 μm, the variability in the measurement greatly increased, and the recovery profile for spots larger than that were too noisy to fit successfully. As the spot size becomes too large, the assumption that the area is uniformly bleached is likely to break down, rendering the analysis model invalid. Therefore, self diffusivity was studied within the pore space of the various scaffolds (30 μm FRAP spot size compared to 160–220 μm pore size).

The normalized self diffusion coefficient divided by the self diffusivity of the solute in liquid, *D*_S_/*D*_S,0_, is often used to assess the impact of the presence of a second phase on diffusivity [42]. For the microporous scaffolds investigated here, the pore size is vastly larger than the size of the dextran molecules used, of the order of a few nanometers [27]. In contrast, in the case of nanoporous hydrogels, the size of the solutes is often comparable to that of the pore size. As a result, the normalized self diffusion coefficient of dextrans in a collagen hydrogel is affected by solute size, decreasing with it [13]. The behavior of the microporous collagen scaffolds considered here is different, as the ratio *D*_S_/*D*_S,0_ did not show a trend with solute molecular weight (Supplementary Fig. 1). This behavior is likely to be due to the larger scale of the morphological features compared with solute size, with the possibility that any small effect of solute size on *D*_S_/*D*_S,0_ may be smaller than the variability in the measurements conducted. Coupled with the inert nature of dextrans [43], which were not seen to bind to the collagen scaffolds (Fig. 3b), this observation allowed for the averaging of the normalized self diffusion coefficients for each structure, irrespective of solutes, to analyze any effect of morphology alone on diffusivity within the pores of the scaffolds.

**Fig. 5:**
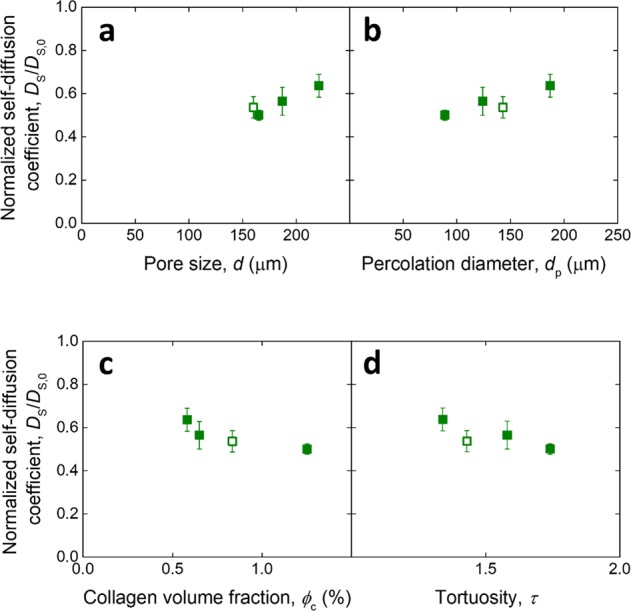
Relationship of self diffusion (averaged over all solute sizes) with scaffold structural parameters. Normalized self diffusion coefficient over the diffusion coefficient of the dextrans in PBS, plotted as a function of **a** pore size, **b** percolation diameter, **c** collagen volume fraction, and **d** tortuosity of the scaffolds. The filled points represent the isotropic structures of varying slurry concentration, while the open points are for the aligned scaffold

Figure 5 shows the normalized self diffusion coefficient (averaged across all solute sizes) plotted as a function of the morphological parameters characterizing the scaffolds. None of the trends were found to be significant (*p* > 0.05 for all fitted line gradients). This is likely to be due to the intrinsic scale-dependency of the measurement, which make properties affecting transport over scales larger than the pore size less relevant within the pore space assessed here. Nevertheless, the self diffusion coefficient was found to be a fraction of *D*_S,0_ (average *D*_S_/*D*_S,0_ = 0.56 ± 0.05), indicating that the presence of the scaffold structure decreases the self diffusivity of the solutes compared to the free fluid. The reason for this phenomenon may be that the presence of the collagen solid phase introduces a hydrodynamic drag on the solutes, whose diffusional jumps are limited by collisions with the pore walls [44]. However, the variability in the structures may be too small here to show an effect on *D*_S_, which overall appears to be only affected by solute size rather than scaffold morphology.

**Fig. 6:**
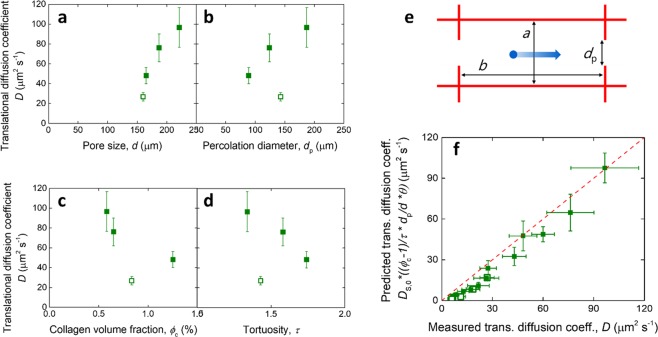
Relationship of translational diffusion with scaffold structural parameters. Translational diffusion coefficient of 4 kDa dextran plotted as a function of **a** pore size, **b** percolation diameter, **c** collagen volume fraction, and **d** tortuosity of the scaffolds. The filled points represent the isotropic structures of varying slurry concentration, while the open point is for the aligned scaffold. **e** Diagram of a scaffold pore defined by its principal pore dimensions *a* and *b*, and its percolation diameter *d*_p_. **f** Relationship between the predicted translational diffusion coefficient, as given in Eq. 4, and the measured translational diffusion coefficient. The dashed line represents the parity line; The filled points represent the isotropic structures of varying slurry concentration, while the open points are for the aligned scaffold

The morphological parameters of the scaffolds do affect diffusion at scales larger than the pore size, i.e. in the case of the translational diffusion coefficient *D* as it is measured in this work. The scaffold structural parameters vary concomitantly upon changes in collagen slurry concentration, so that their separate contribution may not be easily resolved. Figure 6 shows the variation in translational diffusion coefficient with scaffold morphology for one dextran (4 kDa). Within the isotropic structures, *D* increased with pore size *d* and percolation diameter *d*_p_, while it decreased with tortuosity *τ* and collagen volume fraction *ϕ*_c_. However, the fit between aligned and isotropic structures was generally of poor quality, so that an approach capable of concurrently taking into consideration all morphological parameters, as well as solute size, is necessary.

A model originally developed for hydrogels [44] assumes that the decrease in diffusivity (assumed not different between self and translational diffusions) of a solute from its value in unobstructed fluid, *D*_S,0_, is dependent on the probability to find a space for the solute to cross between pores. In the microporous collagen scaffolds, such probability may be approximated by the ratio between percolation diameter and pore size, representing the fraction of the pore wall that is open to the next pore, assuming a rectangular pore cross section (Fig. 5e). This ratio is adjusted for the aspect ratio of the pores, taken as unity for the isotropic structures, and measured as *θ* = *a*/*b* = 0.18 ± 0.07 for the aligned structures, where *a* and *b* represent the shorter and longer radii of the ellipses fit to the pores. Further, the solute path can be thought of being lengthened by the tortuosity of the scaffolds, and limited by the volume fraction of collagen, which results in a non-unity partition coefficient between the fluid outside and inside the scaffold [45]. The resulting predicted translational diffusion coefficient then takes the form:

4 $$D = D_{{\mathrm{S}},0}\frac{{1 - \phi _{\mathrm{c}}}}{\tau }\frac{{d_{\mathrm{p}}}}{d}\theta$$

This predicted coefficient is compared with the measured values in Fig. 6f, where the trend can be seen to broadly follow the reference parity line. Differences between predicted and measured values are likely to be due to the oversimplification of the scaffold geometry, where the pore shape in 3D was previously observed to take that of a tetrakaidekahedron rather than a parallelepiped [11], and multiple pore walls possess openings that are available for diffusion. Nevertheless, Eq. (4) may be adjusted for different pore morphologies that are characteristic of other microporous scaffolds, allowing the prediction of diffusion within those structures.

The relationship between convective fluid transport and diffusive solute transport is not well understood in porous materials used as cell culture substrates [6]. Here, within the isotropic structures, the translational diffusion coefficient was found to vary in a similar way to the fluid permeability of the materials, increasing with *d* and *d*_p_, and decreasing with *τ*, as measured for the same standard set of samples [17]. However, contrary to what was reported for convective transport in these materials, where the fluid permeability of the aligned scaffolds was found to be larger than the isotropic counterparts [17], here it was observed that the translational diffusion coefficient was smaller in the aligned scaffolds (Fig. 4). A structural comparison between these two types of structures made with the same collagen slurry concentration showed that the aligned scaffolds possess a smaller tortuosity and larger percolation diameter in the direction of the alignment, despite a smaller pore size and larger average collagen volume fraction (Table 1). These results suggest that convection and diffusion are differently affected by the complex morphologies of microporous scaffolds, in contrast to what seen in nanoporous hydrogels [6].

In comparison with nanoporous hydrogels, the microporous materials assessed possess a striking capacity to carry solutes, most obvious when considering the translational diffusion coefficient, which impacts the availability of cell nutrients diffusing from a source outside the scaffold. Fitting a power law to the diffusion coefficient results for the 0.5%wt scaffolds, we can predict the diffusion of a protein like albumin, which is metabolized by mammalian cells to extract necessary amino acids [46] and possesses a molecular weight in the range of those considered (≈70 kDa [47]). Our results suggest that albumin would possess an effective room temperature diffusion coefficient of ≈40 μm^2^ s^−1^, much larger than that reported for its diffusion in collagen hydrogels at body temperature (22 μm^2^ s^−1^ at 37 °C) [13].

The values reported in this study can be used to evaluate the influence of structural parameters on the diffusivity of other molecules within microporous scaffolds at different scales, even after adjustments for changes in temperature and cell culture media viscosity. It is critical to note that, in addition to the analysis presented, one may expect that the presence of cell-produced ECM, as well as consumption of nutrients by the cells, will result in a decrease in the overall transport depth [43, 48]. Similarly, our analysis does not take into consideration electrostatic [49] or osmotic [50] effects within the scaffolds, which were found to impact fluid transport and so may also affect solute transport. However, the open nature of the freeze-dried scaffolds will likely make them less affected by these factors compared with denser hydrogels. For these reasons, the microporous materials considered here may be more suitable than nanoporous hydrogels to allow 3D culture of cells at large depths.

## Conclusions

The studies conducted here show that diffusion of macromolecules within microporous tissue engineering scaffolds is scale-dependent, with different structural contributions to transport depending on the distance from the source of solutes. This scale dependence of the diffusion is interesting, as from a practical point of view, the translational diffusion coefficient dominates the transport of nutrients when the source is distant, outside the scaffold during in vitro culture, while the self diffusion coefficient dominates the transport from de novo vasculature within the pores of the scaffold after implantation in vivo, with the two types of diffusion differently affected by the scaffold morphology. The results reported here, therefore, provide insight into the development of scaffolds with custom concentration gradient profiles and increased efficacy as platforms for 3D cell culture.

## Supplementary information

Supplementary Information
