# Supplementary material for: Scale and structure dependent solute diffusivity within microporous tissue engineering scaffolds
Source: J Mater Sci Mater Med. 2020 May 4;31(5):46. doi: 10.1007/s10856-020-06381-x (PMC7198636; doi:10.1007/s10856-020-06381-x)
Supplement: Supplementary file 1 — Supplementary Information [file 10856_2020_6381_MOESM1_ESM.docx]

**SUPPLEMENTARY INFORMATION**

**
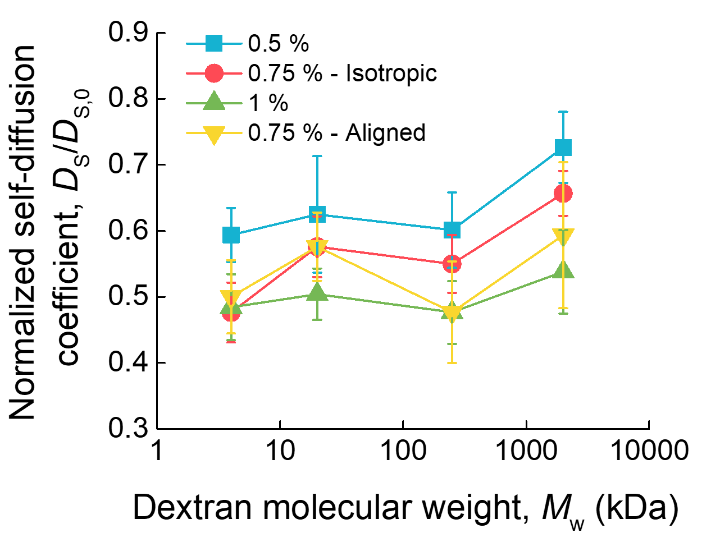
**

**Figure S1.** Self diffusion coefficients of dextran of varying molecular weight normalized over the specific self diffusion coefficient of the molecules in PBS, within the collagen scaffold structures assessed.


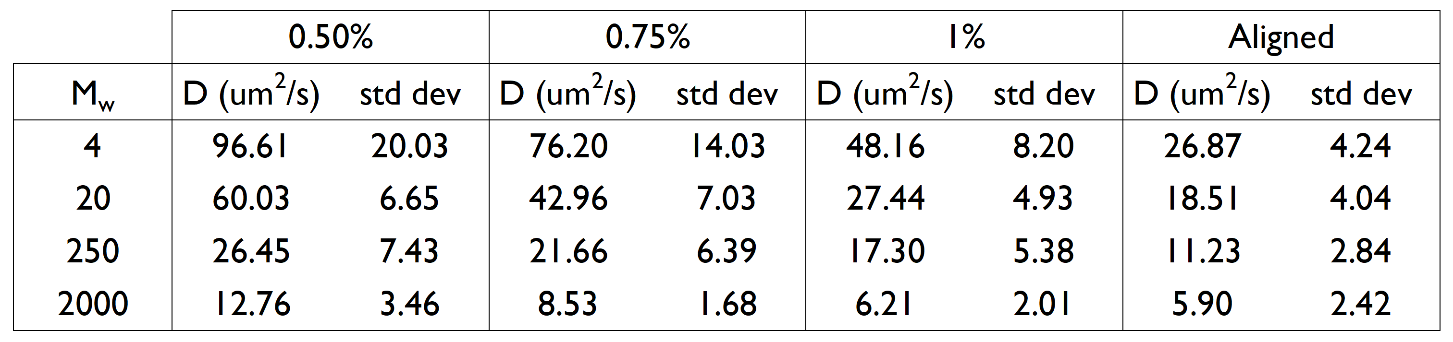


**Table S1.** Average and standard deviation of translational diffusion coefficients of dextran as a function of molecular weight in the collagen scaffold structures assessed.


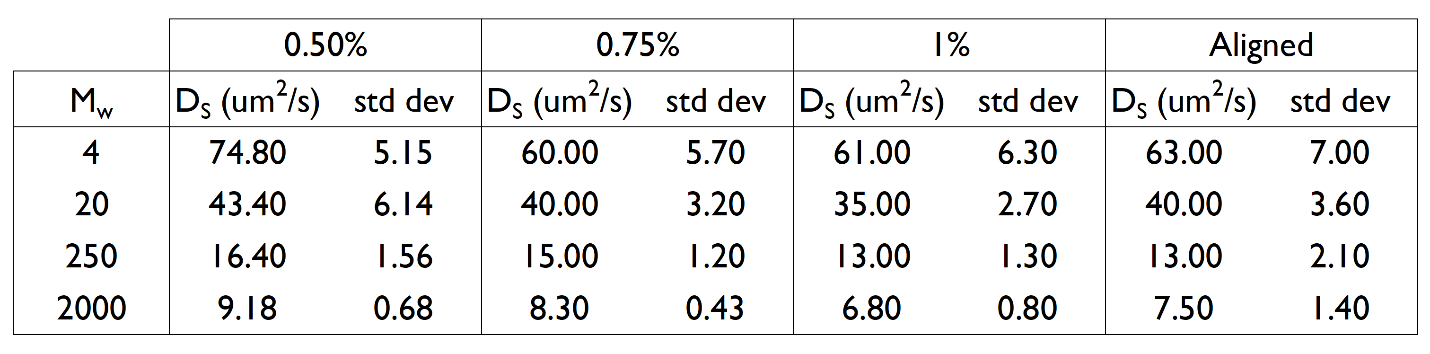


**Table S2.** Average and standard deviation of self diffusion coefficients of dextran as a function of molecular weight in the collagen scaffold structures assessed.
